# Time-of-Day Could Affect Cognitive Screening Performance in Older Patients with TIA and Stroke

**DOI:** 10.1159/000456673

**Published:** 2017-03-21

**Authors:** Sara Mazzucco, Linxin Li, Maria A. Tuna, Sarah T. Pendlebury, Rhoda Frost, Rose Wharton, Peter M. Rothwell, on behalf of the Oxford Vascular Study

**Affiliations:** Centre for Prevention of Stroke and Dementia, Nuffield Department of Clinical Neurosciences, John Radcliffe Hospital, University of Oxford, Oxford, UK

**Keywords:** Cerebrovascular disease, Cognitive impairment, Aging, Circadian rhythm

## Abstract

**Background and Purpose:**

The impact of time-of-day on the cognitive performance of older patients with limited cognitive reserve after a transient ischemic attack (TIA) or stroke, and on short cognitive tests, such as the Montreal Cognitive Assessment (MoCA), is unknown. We retrospectively studied whether morning versus afternoon assessment might affect the classification of patients aged 70 or older as severe (SCI), mild (MCI), and no (NCI) cognitive impairment by the MoCA.

**Methods:**

Morning (12 p.m. or earlier) versus afternoon (later than 12 p.m.) proportions of SCI (MoCA score <20), MCI (MoCA score 25-20) and NCI (MoCA score ≥26) were compared in a cohort of patients aged ≥70, attending a rapid-access TIA/stroke clinic.

**Results:**

Of 278 patients, 113 (40.6%) were tested in the morning and 165 (59.4%) in the afternoon. The proportion with SCI was greater in the afternoon than in the morning (10.9 vs. 1.8%, respectively, *p* = 0.004), with no difference in age, education, diagnosis, disability, or vascular risk factors.

**Conclusions:**

Time-of-day appears to affect cognitive performance of older patients after they undergo TIA and minor stroke. If our cross-sectional findings are confirmed in cross-over studies with repeated testing, timing of assessments should be considered in clinical practice and in research studies.

## Introduction

Many trials look at the effect of stroke and stroke-preventive treatments on cognitive trajectory and on the transition from mild cognitive impairment (MCI) to dementia [1]. The reliable distinction between the 2 cognitive states and its reproducibility is therefore important [2]. Differences between diagnostic criteria for MCI can have an impact on the prevalence and rate of progression to dementia [3], but operational differences within well-established criteria can also result in significant variation in MCI estimates [2]. For example, it is known that there is diurnal variation in cognitive abilities, and that after 60, cognitive performance tends to worsen through the day [4, 5]. In patients with limited cognitive reserve, such as those with MCI, this variation might be clinically relevant. However, according to the extent of our knowledge, no studies on stroke or transient ischemic attack (TIA) have reported on the consistency of timing of cognitive screening, and there are no studies on the impact of time-of-day on cognitive performance as assessed by short cognitive tests, which are increasingly used both in clinical trials and in clinical practice to measure cognitive outcomes [2].

Among short cognitive tests used in patients with cerebrovascular disease, the Montreal Cognitive Assessment (MoCA) test has proved to be one of the most sensitive and specific for MCI, and it incorporates executive and attentional tasks suitable for vascular cognitive impairment [2, 6, 7]. In a cohort of patients aged ≥70 attending a rapid-access TIA/stroke clinic, we did a retrospective cross-sectional study to determine whether there was any evidence to suggest that time-of-day of assessment might affect the categorisation of patients as “severe cognitive impairment” (SCI) as compared to “MCI” or “no cognitive impairment” (NCI) by the MoCA.

## Materials and Methods

### Participants

Consecutive eligible patients aged ≥70, followed-up 1 month after attending the Oxford Vascular Study (OXVASC) rapid-access TIA/stroke clinic between November 2011 and January 2016 were enrolled in this study. The OXVASC Study is an ongoing population-based study on the incidence and outcome of all acute vascular events in a population of 92,728 individuals registered with 100 primary care physicians in 9 practices in Oxfordshire, United Kingdom. Multiple methods of ascertainment are used for patients with TIA or stroke [8], including a daily, rapid-access TIA/stroke clinic, to which participating physicians and the local emergency department refer all individuals with suspected TIA or minor stroke [9].

### Procedures

Patients were assessed and treated in the acute phase by a neurologist or stroke physician and all presentations and investigations were reviewed by the senior study neurologist (P.M.R.) as discussed elsewhere [10]; the MoCA was administered at 1 month follow-up to consenting patients. On the MoCA, SCI was defined as a score <20, MCI as a score between 25 and 20, while NCI was defined as a score ≥26 [6, 11, 12].

The OXVASC study was approved by the local ethics committee and consent was obtained from all participants.

### Statistical Analysis

Consecutive patients aged ≥70 who had been able to undergo cognitive assessment after presumed TIA or minor stroke (NIHSS ≤3) were included in the analysis if they had no pre-existing clinical diagnosis of dementia and had no disabling neurological deficit due to previous stroke.

Demographic and clinical characteristics and risk factors of patients in the “morning” and in the “afternoon” group were compared using χ^2^ test or ANOVA as appropriate. Proportions of patients with SCI, MCI and NCI assessed in the morning (12 p.m. or earlier) versus afternoon (later than 12 p.m.) were analysed using χ^2^ test.

## Results

Of 279 patients aged ≥70 undergoing cognitive screening with the MoCA 1 month after attending a rapid-access TIA/stroke clinic, one was excluded from the analysis because the time of the cognitive testing was not recorded, leaving 278 patients in the final analysis. Of these, 113 (40.6%) were tested in the morning and 165 (59.4%) in the afternoon. The median time of assessment in the morning was 10.55 a.m. (interquartile range [IQR] 10.15 a.m.-11.30 a.m.), while in the afternoon it was 1.49 p.m. (IQR 12.44 p.m.-2.53 p.m.).

The “morning” and the “afternoon” groups did not differ significantly in terms of age, diagnosis, education, modified Rankin Scale, and risk factors (Table 1).

Overall, 163 patients were classified as NCI, 95 as MCI and 20 as SCI. While there was no significant difference in morning vs. afternoon proportion of NCI (*p* = 0.95) or MCI (*p* = 0.1), patients assessed in the afternoon were significantly more likely to display SCI than patients who were assessed in the morning (*p* = 0.004; Table 2).

## Discussion

To our knowledge, this is the first study to explore the effect of time-of-day on cognitive performance as assessed by short cognitive tests, such as the MoCA, and the first study in older patients with TIA and stroke. Short tests are increasingly used not only in clinical practice to follow-up patients with cognitive impairment but also in large multicentre clinical trials both as screening tools and as cognitive outcomes [1]. To our knowledge, previous clinical trial protocols have not required consistent timing of cognitive assessments.

In our study, patients tested in the afternoon were more likely to be classified as SCI as compared to patients tested in the morning. This could be consistent with our hypothesis that older patients with limited cognitive reserve might be more susceptible to cognitive fluctuations triggered by environmental factors, including time-of-day. Previous studies have suggested that in older subjects, cognitive performance tends to worsen through the day, while in younger people it is worse early in the morning and improves along the day [5].

Our study has limitations. First, our analysis was purely cross-sectional and our findings need to be confirmed through cross-over studies with repeated assessments at different times of day. Our patients had had a prior cognitive assessment in the acute phase after their TIA or stroke, but this had usually been in the morning only and was also confounded by acute-phase effects [10, 13], undermining the interpretation of any differences from the 1-month follow-up assessments reported here. However, our findings have highlighted the potentially important but neglected the issue of the timing of assessment, and the need for further prospective studies required to formally test them. Second, we were able to study diurnal variation in cognition only during office hours (9 a.m.-5 p.m.). Although this period is probably most relevant in relation to clinical assessments and research studies, reduced cognitive performance in the evening could be a greater clinical problem.

In conclusion, time-of-day appears to affect cognitive performance of older patients after TIA and minor stroke. If our findings are confirmed in cross-over studies with repeated assessments, timing of testing would be particularly important in implementing research protocols in studies with repeated cognitive assessments after TIA or stroke; these are studies that predominantly involve older patients.

## Sources of Funding for the Study

The Oxford Vascular Study has been funded by the Wellcome Trust, Wolfson Foundation, UK Stroke Association, British Heart Foundation, National Institute of Health Research (NIHR), Dunhill Medical Trust and the NIHR Biomedical Research Centre, Oxford. Dr. L. Li was funded by the China Scholarship Council. Dr. M.A. Tuna was funded by the Gulbenkian Foundation Doctoral Programme for Physicians. Prof. S.T. Pendlebury was funded by the Oxford NIHR Biomedical Research Centre. Prof. P.M. Rothwell is in receipt of an NIHR Senior Investigator Award and a Wellcome Trust Senior Investigator Award.

## Disclosure Statement

The authors have no disclosures to make.

## Figures and Tables

**Table 1:** Characteristics of 278 patients assessed in the morning versus afternoon

	Morning (*n* = 113)	Afternoon (*n* = 165)	*p* value
Age, years, mean (SD)	78.12 (5.65)	78.19 (5.51)	0.925
mRS at 1 month >1	34 (30.1)	46 (29.1)	0.483
Low education	62 (54.9)	95 (57.6)	0.373
Diagnosis, *n* (%)			
TIA	76 (67.3)	101 (61.2)	0.352
Stroke	30 (26.5)	46 (27.9)	
Non–vascular	7 (6.2)	18 (10.9)	
Risk factors, *n* (%)			
History of hypertension	68 (60.2)	112 (67.9)	0.117
History of diabetes	18 (15.9)	24 (14.5)	0.439
History of hyperlipidaemia	42 (37.5)	57 (35.4)	0.410
History of smoking	64 (57.1)	78 (48.1)	0.090
Atrial fibrillation	12 (10.6)	28 (17)	0.094

Data are number (%) unless otherwise stated. mRS, modified Rankin Scale; Low education, education ≤12 years.

**Table 2:** Cognitive performance according to the time-of-day of assessment in 278 patients aged ≥70

Study population	Morning, *n* (%)	Afternoon, *n* (%)	*p* value
NCI (MoCA ≥26)	66 (58.4)	97 (58.8)	0.95
MCI (MoCA 25–20)	45 (39.8)	50 (30.3)	0.1
SCI (MoCA <20)	2 (1.8)	18 (10.9)	0.004
Total	113 (100)	165 (100)	0.008

NCI, no cognitive impairment; MCI, mild cognitive impairment; SCI, severe cognitive impairment.
